# South West Orthopaedic Club

**Published:** 1990-06

**Authors:** 


### South West Orthopaedic Club

### Spring Meeting in Cheltenham 1989

### GORETEX PROSTHETIC CRUCIATE LIGAMENT RECONSTRUCTION

**Philip V. Seal**, Hereford and Oswestry

Cruciate ligament rupture, anterior in the main, is a relatively common injury. The management of this has taxed orthopaedic surgeons for pretty well the whole of the present century. Management continues to be dificult and debatable. Prosthetic replacement has become more popular over the past few years. Long term results are awaited.

**Clinical material**: Since February 1985 (4.2 years) 75 implants have been inserted. 62 Mark I ligaments and since January 1989, 13 Mark II ligaments.

This paper discusses the experience with the 62 Mark I ligaments.

56 male, 61 ACL—in all but one, giving way, either at sport or at leisure was the predominant presenting symptom. Correlation between the laxity signs and instability symptoms were not as direct as previously thought.

**1. Fixation**

Screw through eyelet appears to be very secure. No failure has occurred. No significant bone growth has been noted from the bone tunnels into the ligment. This may occur peripherally. With time, dense fibrous fixation occurs at the exits of the tunnels, affording very secure fixation.

**2. Biology**

Short lasting effusions occurred in 11 (17%), only 2 more tnan once. No chronic synovitis. PTFE used as a cruciate ligament is not completely bio-inert but biological problems at present are not significant .

**3. Strength**

The Gore ligament is in general terms, twice as strong as a normal cruciate ligament. 5 (8%) have failed, 1 clearly at the deep exit of the femoral tunnel and 4 within the knee. Only 1 occurred playing violent sport, 4 with no specific injury. Abrasion within the intercondylar notch or at the deep exit of the femoral tunnel is the most likely explanation. Undertaking notchplasty with attention directed to the lateral aspect of the notch and also to the superior aspect in full extension is vitally important.

The Mark II ligament has been designed to try and improve abrasion characteristics.

**4. Elasticity**

In the majority of instances, laxity signs were abolished at surgery. At follow up, there was some increase in the laxity signs. This could be due to the following:
(a) creep in the material
(b) soft tissue necrosis between the ligament and the posterior aspect of the femur
(c) hidden rupture

One patient has demonstrated laxity causing recurrence ot symptoms but no significant rupture of the prosthesis.

7 (11%) have been revised, 4 for rupture, 2 being too tight (blocking terminal extension) and 1 which became slack without rupture.

Immediate post operative problems were minimal. No infection has occurred and subjectively, many of the patients are pleased with the outcome and are able to return to sport.

**Conclusions**: Goretex prosthetic reconstruction of ruptured cruciate ligaments seems with a 4.2 year follow up, to be a satisfactory mode of treatment for significantly disabled patients. Further, continuing careful review is necessary over the next 5 to 10 years, to fully assess this mode of treatment.

### THE UNCEMENTED P.C.A. UNICOMPARTMENTAL REPLACEMENT FOR OSTEOARTHRITIS OF THE KNEE

**P. A. Magnussen**, Bristol Royal Infirmary, Bristol, England
**R. J. Bartlett**, Austin Hospital, Melbourne, Australia

Unicompartmental joint replacement for osteoarthritis of the knee has remained controversial. We report the early results of 50 uncemented P.C.A. unicompartmental replacements for osteoarthritis of the knee, performed by one surgeon between 1985 and 1987, and studied prospectively. Forty-one medial prostheses in 37 patients and 9 lateral prostheses in 9 patients were reviewed on average 2 years after surgery. At review, a subjective opinion and an objective result, using the Bristol Knee Score was obtained.

In the medial joint replacement group, 85 per cent had subjectively a good result, 5 per cent fair and 10 per cent poor. Objectively, 92 per cent were good and 8 per cent fair. All the lateral joint replacements had a good result. This improvement was mainly due to pain relief, with pre-operative range of movement being maintained. Of the 4 failures, two were due to technical error, one a failure of patient selection and the last, an unexplained synovitis.

The early results for the uncemented P.C.A. unicompartmental knee replacement are encouraging, with a good result in greater than 85 per cent and a minimal complication rate.

### TOTAL HIP REPLACEMENT IN THE EIGHTY YEAR OLD

**D. P. Newington and G. C. Bannister**, Winford Orthopaedic Hospital, Bristol

With current population changes, the number of patients of 80 or more years, who present for primary total hip replacement is increasing. To evaluate the procedure in this age group, 107 octogenarians, who received primary total hip replacement between 1978 and 1985 were reviewed. Data was obtained from hospital records, surviving patients, first degree relatives or Family Practitioners, and the outcome was assessed using a modified Harris Hip Score.

Acute dislocation occurred in 15% of patients and became chronic in 2/3 of these. This was 7 times greater than the general dislocation rate in Bristol and bore no relation to the surgical approach or the prosthesis used.

There were 5 fractures of the femoral shaft which was 4 times higher than the norm. In addition, there were 4 perforations of the femoral shaft and 1 acetabular fracture. These operative complications were associated with a particularly poor outcome and hospital admission of 5 months' duration.

Perioperative mortality was 4% and morbidity significantly greater in patients who sustained a technical complication. In 7 patients with either fracture or dislocation, multiple medical problems ensued.

The average admission was 37 days. However, patients who sustained fractures remained for 149 days and dislocations for 71. By contrast, the bed occupancy of uncomplicated cases was 27 days.

Although the final outcome correlated with the severity of symptoms before surgery, it was not possible to identify those who fared badly prospectively. 75% of patients achieved satisfactory results and this related more to relief of pain than improvement of mobility.

Patients over 80 who undergo primary hip arthroplasty experience special problems. Their particular needs are an enhanced awareness of dislocation, extreme care over the technical aspects of the procedure and prompt attention to ensuing medical problems. Careful explanation of the risk of complication to both patient and relatives may help to temper the disappointment of an unsatisfactory result.

### A COMPARISON OF TOTAL HIP REPLACEMENT, DOUBLE CUP ARTHROPLASTY AND OSTEOTOMY IN PATIENTS UNDER THE AGE OF 50

**N. R. Boeree, A. Parry, G. C. Bannister and P. H. Roberts**
Winford Orthopaedic Hospital, Bristol

93 procedures in 76 patients under 50 with arthritis of the hip were assessed clincially by the Harris hip score and by survivorship analysis. 43 cemented total hip replacements were carried out for 31 patients, 23 double cup arthroplasties for 26 cases and 27 osteotomies for 26 young arthritic patients. Follow-up was for a mean of 1, 6.9 and 6.3 years respectively.

The mean Harris hip score was 92 in primary hip replacements, 62 in double cup arthroplasties and 73 in osteotomies ($p<0.001$). Failures in each group that were revised to total hip replacement scored 93 after total hip replacement, 77 after double cup arthroplasty and 63 after osteotomy ($p<0.01$).

After 7 years one (2.3%) total hip replacement, 10 (42%) double cup arthroplasties and 6 (22%) osteotomies had been revised. Survivorship analysis of the entire sample indicated an annual risk of failure of 1.8% for total hip replacement, 5.2% for double cup arthroplasty and 5.9% for osteotomy.

Primary total hip replacement appeared to give superior and longer lasting results than double cup arthroplasty or osteotomy. The functional quality of hip replacement after double cup arthroplasty or osteotomy was inferior to that of primary or revised hip replacement.

There is no perfect solution to the problem of the young arthritic hip but primary hip replacement seems the best of the available alternatives.

### BONE BANKING IN THE ANTIPODES WHAT CAN BRISTOL LEARN FROM BRISBANE?

**D. Eastwood**
Bristol Royal Infirmary

Many orthopaedic procedures require bone graft and whilst autograft has been used successfully for many years the supply is often limited and associated with considerable morbidity; allograft bone can be harvested from both living donors and multi-organ donors and then stored in a Bone Bank until required.

The Princess Alexandra Hospital in Brisbane set up a Hospital Bone Bank 18 months ago and has harvested bone from 41 elective operations and from 9 cadaveric donors. Initially, many of the specimens were discarded due to bacterial contamination but with improvement in harvesting technique this contamination rate has reduced.

Twenty-four patients (mainly undergoing revision arthroplasty) have received fresh frozen allograft bone in mulch and block form. Six patients have received fresh osteochondral allografts to replace damaged joint surfaces and restore normal joint anatomy. After a short period of follow-up, there have been no cases of infection or rejection.

A Bone Bank does not have to be a large commercially orientated organisation extending over half a continent; it could be run successfully at District level to the benefit of both the South West Orthopaedic Surgeons and their patients.

